# Clinical Profile and Management of Maxillofacial Injuries in a Tertiary Care Hospital in North India: A Prospective Observational Study

**DOI:** 10.7759/cureus.103594

**Published:** 2026-02-14

**Authors:** Marimuthu P, Mohd. Kalim Ansari, Ghulam S Hashmi, Keerthy Ajay Kumar, Anupriya L, Saima Y Khan

**Affiliations:** 1 Oral and Maxillofacial Surgery, Dr. Ziauddin Ahmad Dental College and Hospital, Aligarh Muslim University, Aligarh, IND; 2 Anatomy, Jawaharlal Nehru Medical College, Aligarh Muslim University, Aligarh, IND; 3 Pediatric and Preventive Dentistry, Dr. Ziauddin Ahmad Dental College and Hospital, Aligarh Muslim University, Aligarh, IND

**Keywords:** epidemiology, facial fractures, management strategies, maxillofacial injuries, road traffic accident

## Abstract

Background: Maxillofacial injuries significantly affect facial aesthetics, functional capacity, and quality of life. With the growing societal emphasis on physical appearance, a thorough understanding of the etiology, clinical presentation, and management of these injuries has become important.

Methods: A two-year prospective study was conducted from December 31, 2020, to October 31, 2022 (22 months) at the Department of Oral and Maxillofacial Surgery, Dr. Ziauddin Ahmed Dental College, and the Emergency Department of Jawaharlal Nehru Medical College and Hospital, Aligarh, Uttar Pradesh. Patient demographics, etiology of injury, anatomical site, associated injuries, and treatment modalities were recorded. Data analysis was performed using IBM SPSS Statistics for Windows, Version 20.0 (Released 2011; IBM Corp., Armonk, NY, USA). As this was a descriptive study, results were summarized using descriptive statistics such as frequencies and percentages.

Results: Among 400 patients, the mean age was 25.9 ± 1.99 years. Males constituted 80% (n = 320), yielding a 4:1 male-to-female ratio. The most affected age group was 21-30 years (41.2%, n = 165). Road traffic accidents (RTAs) were the predominant cause (77.7%, n = 275), followed by falls (18%, n = 44) and assaults (4%, n = 16). Middle-third fractures were most frequent (53%, n = 212), followed by mandibular fractures (30%, n = 120). Condylar fractures were the most common mandibular injury (28.2%, n = 113), while zygomaticomaxillary complex (ZMC) fractures were the most prevalent midfacial injuries (28%, n = 112). Head injuries (63.2%, n = 148) were the most common associated trauma. Open reduction and internal fixation (ORIF) was performed in 61.7% (n = 247) of cases.

Conclusion: Maxillofacial injuries disproportionately occur in young male individuals, with RTAs (most notably those involving motorcycles and other two-wheeled vehicles) constituting the principal etiological factor. In clinical practice, this necessitates heightened preparedness for immediate airway stabilization, definitive management of maxillofacial fractures, and integration of multidisciplinary trauma protocols. Furthermore, active participation of clinicians in advocating protective helmet usage, reinforcing community-based injury prevention programs, and facilitating streamlined referral systems may contribute to decreasing trauma burden, improving treatment outcomes, and limiting long-term functional and aesthetic sequelae.

## Introduction

Injuries affecting the facial region can have a deep and long-term impact on an individual’s quality of life, especially in today’s society, where facial appearance and aesthetics carry significant social importance [1]. Apart from cosmetic concerns, the face is essential for daily functions, such as eating, speaking, breathing, and vision, and also plays a key role in communication and personal identity. Trauma to this region can therefore lead not only to physical disability but also to emotional and psychological stress. Patients with facial injuries may experience reduced self-confidence, difficulty in social interactions, and long-term psychological effects, highlighting the importance of effective management and rehabilitation.

The treatment of maxillofacial trauma continues to be a major clinical challenge worldwide because these injuries are frequently associated with functional impairment, visible deformity, and high treatment costs [2]. The facial skeleton has a complex structure and lies in close proximity to vital organs such as the brain, eyes, and airway, making diagnosis and treatment demanding. In many cases, successful management requires coordination between multiple specialties, including maxillofacial surgery, neurosurgery, ophthalmology, and emergency medicine. In developing regions, challenges such as delayed hospital presentation, limited healthcare resources, and uneven access to specialized care may further influence treatment outcomes.

Maxillofacial fractures are commonly encountered in trauma practice and may occur as isolated injuries or along with severe injuries to the head, spine, or limbs, often requiring urgent evaluation and intervention [3]. Early diagnosis and appropriate treatment are essential to prevent serious complications and to restore both function and appearance. The pattern and severity of fractures are often related to the type and intensity of trauma, making epidemiological understanding important for planning trauma care services and preventive strategies.

Social behavior, infrastructure, and lifestyle patterns strongly influence the occurrence of facial trauma in the studied population [4]. Increasing vehicle use, poor adherence to traffic safety measures, and risk-taking behavior contribute significantly to the incidence of maxillofacial injuries. Additional factors such as alcohol use, irregular use of protective equipment, and road safety conditions may further affect trauma patterns in certain areas.

Despite the clinical importance of maxillofacial trauma, region-specific data on injury patterns, epidemiology, and treatment outcomes remain limited. High dependence on two-wheeler transportation, inconsistent helmet use, and variations in the availability of specialized trauma services may influence both the occurrence and management of these injuries. Understanding these regional characteristics is important for improving trauma care delivery and developing targeted preventive strategies.

Therefore, this prospective study was conducted to evaluate age-wise distribution, incidence, fracture characteristics, and treatment approaches in patients presenting with maxillofacial trauma. The findings were also compared with national and international data to better understand regional trends and identify gaps in trauma care and prevention.

## Materials and methods

A 22-month prospective observational study was conducted in the Department of Oral and Maxillofacial Surgery, Dr. Ziauddin Ahmed Dental College, and the Emergency Department of Jawaharlal Nehru Medical College and Hospital, Aligarh, Uttar Pradesh, from December 2020 to October 2022 after obtaining approval (Ref. No.: IECJNMC/438; approval date: 04/09/2021).

A data collection proforma was created to record all required patient and clinical details in a uniform manner. It was prepared after reviewing relevant literature and was checked by senior clinicians to ensure that the format was clear, appropriate, and suitable for collecting accurate study data. Patients were evaluated by demographic data, cause of injury, place of injury, pattern of maxillofacial injury, associated injuries, and treatment plan.

A total of 400 patients were included in this prospective observational descriptive study. The sample size was determined based on feasibility, institutional caseload, and the need for adequate statistical representation, rather than on a formal power calculation.

The minimum required sample size was estimated using the standard formula for proportions, where Z = 1.96 (95% CI), p = 0.5 (assumed prevalence to maximize sample size), q = 1 − p, and d = 0.05. This yielded a minimum sample size of approximately 384, which was rounded to 400 to improve data completeness and reliability.

The modes of injuries were classified as road traffic accidents (RTAs), falls from height, assault, sports-related, occupational-related, and others (animal attack, explosive injury, etc.). Fractures were assessed according to the location, that is, upper third, middle third, lower third, and a combination of both middle and lower third. The upper third face includes the frontal bone and frontal sinus injuries. Midfacial sites were classified as maxilla, zygoma, naso-orbito-ethmoid, isolated zygomatic arch, orbital floor, and nasal. The midface fractures were also classified according to Le Fort classification. Sites of mandibular fractures were classified as symphysis, parasymphysis, body, angle, ramus, condyle, coronoid, and dentoalveolar.

Patients presenting to the Emergency and Trauma Centre of Jawaharlal Nehru Medical College with maxillofacial fractures, including those admitted or treated on an outpatient basis in the Department of Oral and Maxillofacial Surgery, Dr. Ziauddin Ahmed Dental College and Hospital, were included. Patients with old facial fractures referred for reconstructive surgery and those who did not provide informed consent were excluded.

Statistical analysis

The collected data were first recorded in Microsoft Excel 2010 (Microsoft Corp., Redmond, WA, USA) and subsequently processed using IBM SPSS Statistics for Windows, Version 20.0 (Released 2011; IBM Corp., Armonk, NY, USA). The analysis was limited to descriptive statistical methods. Demographic and clinical variables, such as age, sex, etiology of injury, and fracture distribution, were presented in terms of frequency counts and percentage values.

## Results

The present study followed a descriptive design, and categorical variables were summarized using frequencies and proportions. Demographic and clinical characteristics, including gender distribution, age categories, mechanism of injury, fracture patterns, and treatment methods, were described to outline the overall trend within the study population. RTAs constituted the most common cause of injury among male patients. Differences in fracture patterns were observed across various age categories. The distribution of treatment approaches did not show noticeable variation between male and female patients.

**Figure 1:**
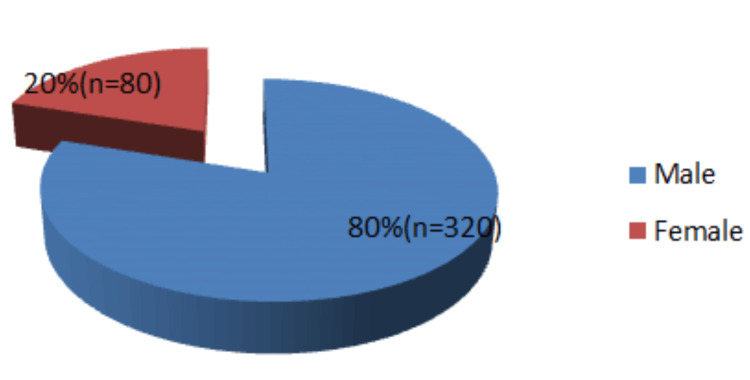
Gender distribution of the study population (N = 400)

A total of 400 patients with maxillofacial injuries were included, with ages ranging from 3 to 85 years (mean age: 25.9±1.99 years). Males constituted 80% (n = 320) and females 20% (n = 80), with an overall male-to-female ratio of 4:1. The most affected age group was 21-30 years (41.2%), and male predominance was noted across all age groups (Figure 1).

**Figure 2:**
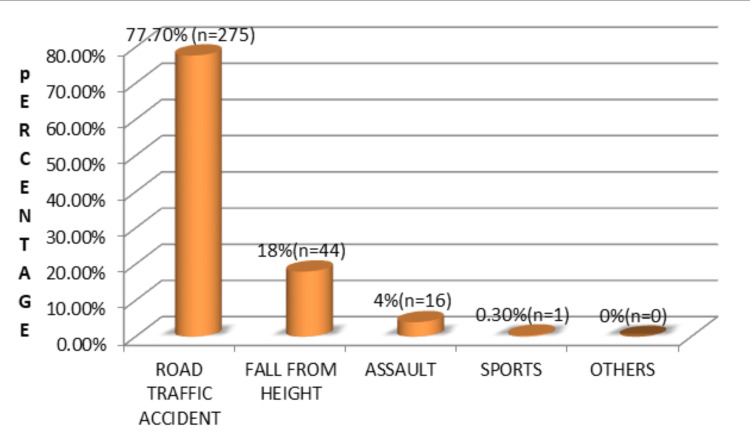
Etiology of maxillofacial injuries (N = 400)

RTAs were the leading cause of injury in both genders, with two-wheelers being the most commonly involved vehicles (Figure 2). Drivers were more frequently affected compared to passengers and pedestrians.

**Figure 3:**
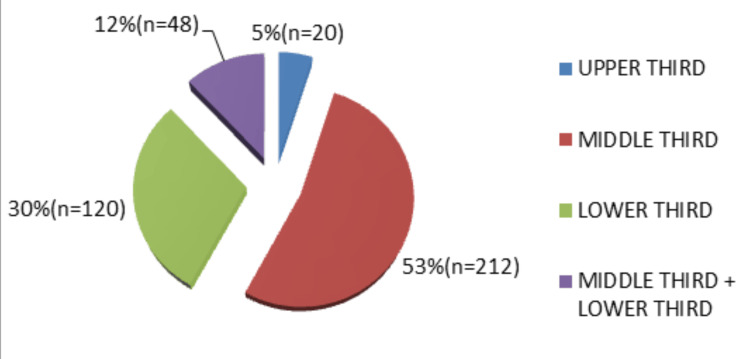
Distribution of maxillofacial fractures by facial third (N = 400) Middle-third fractures were the most common (53%, n = 212), followed by the lower-third (30%, n = 120), combined middle and lower third (12%, n = 48), and upper-third fractures (5%, n = 20).

**Figure 4:**
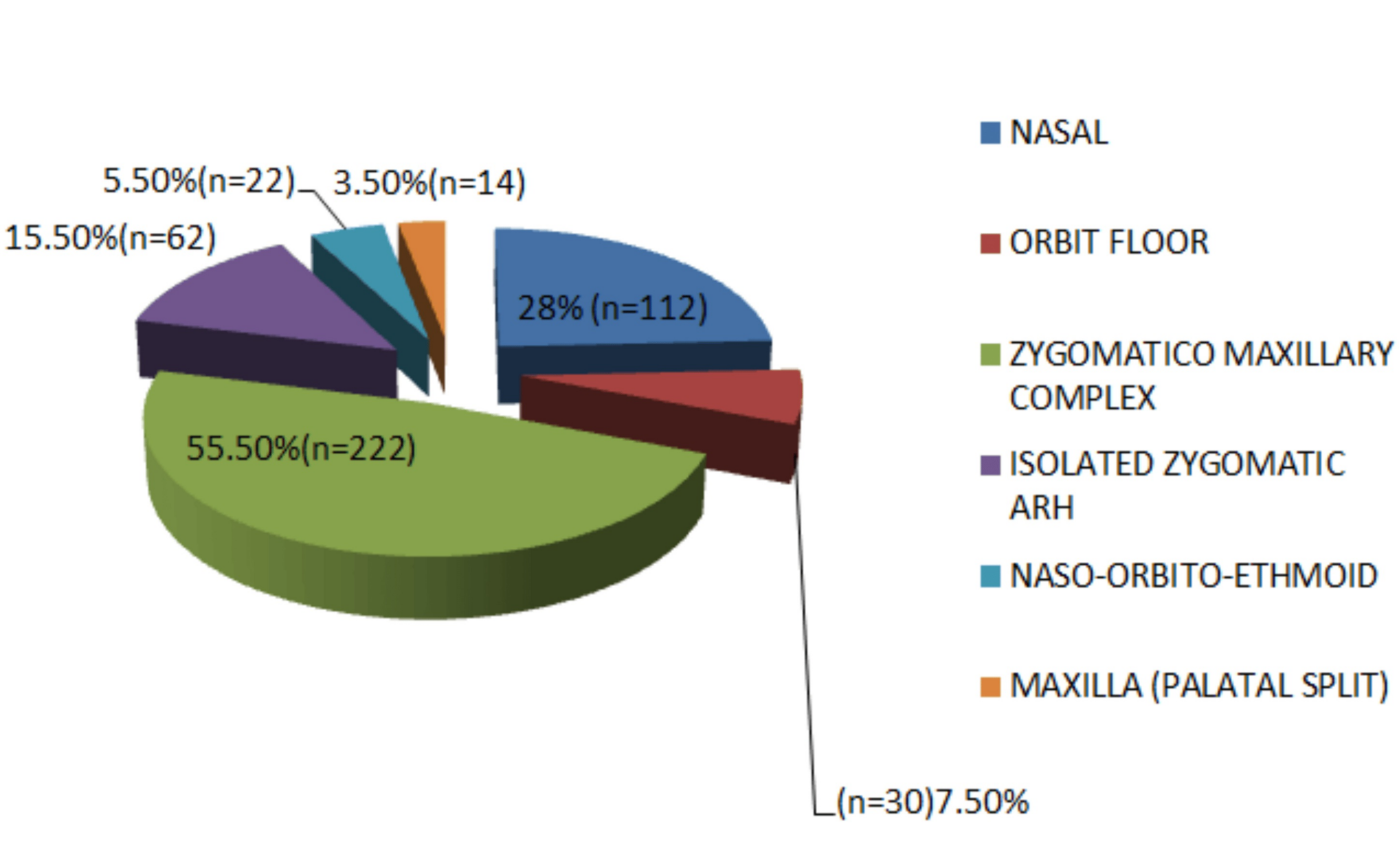
Distribution of middle-third facial fractures by anatomical site (N = 400) Zygomaticomaxillary complex fractures were the most frequent (55.5%, n = 222), followed by nasal (28%, n = 112), isolated zygomatic arch (15.5%, n = 62), orbital floor (7.5%, n = 30), naso-orbito-ethmoid (5.5%, n = 22), and maxillary palatal split fractures (3.5%, n = 14).

**Figure 5:**
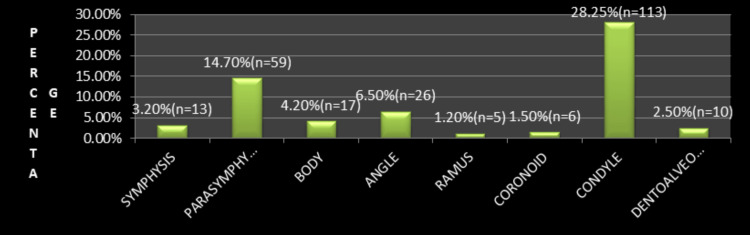
Distribution of mandibular fracture sites (N = 400) Condylar fractures were the most common (28.25%, n = 113), followed by parasymphysis (14.7%, n = 59), angle (6.5%, n = 26), body (4.2%, n = 17), symphysis (3.2%, n = 13), dentoalveolar (2.5%, n = 10), coronoid (1.5%, n = 6), and ramus fractures (1.2%, n = 5).

Midface fractures were the most frequently observed (53%), followed by mandibular fractures (30%), combined midface and mandibular fractures (12%), and upper third facial fractures (5%). Among midface injuries, zygomaticomaxillary complex (ZMC) fractures were most common, followed by nasal bone fractures. In mandibular fractures, the condyle was the most frequently involved site, followed by the parasymphysis and angle regions (Figures 3-5).

**Figure 6:**
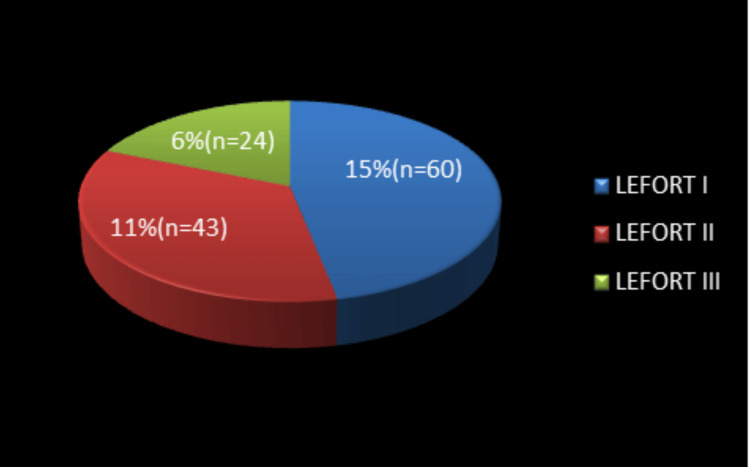
Distribution of Le Fort fracture patterns (N = 400) Le Fort I fractures accounted for 15% (n = 60), followed by Le Fort II (11%, n = 43) and Le Fort III fractures (6%, n = 24).

**Figure 7:**
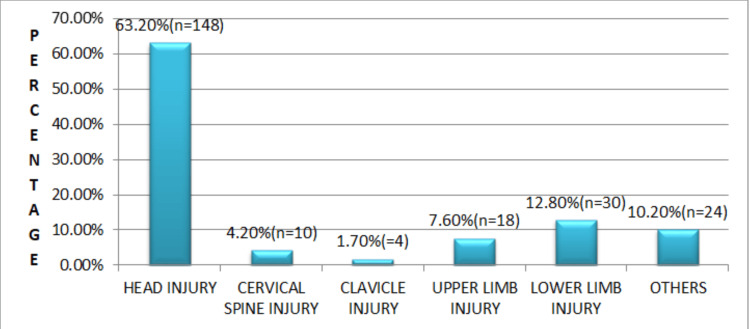
Distribution of associated injuries in the study population (N = 400) Head injuries were the most frequent (63.2%, n = 148), followed by lower limb (12.8%, n = 30), other injuries (10.2%, n = 24), upper limb (7.6%, n = 18), cervical spine (4.2%, n = 10), and clavicle injuries (1.7%, n = 4).

Le Fort I fractures were the most common Le Fort pattern, followed by Le Fort II and Le Fort III fractures. Associated injuries were most commonly head injuries (63.2%), followed by lower limb injuries and thoracoabdominal trauma (Figures 6, 7). 

**Figure 8:**
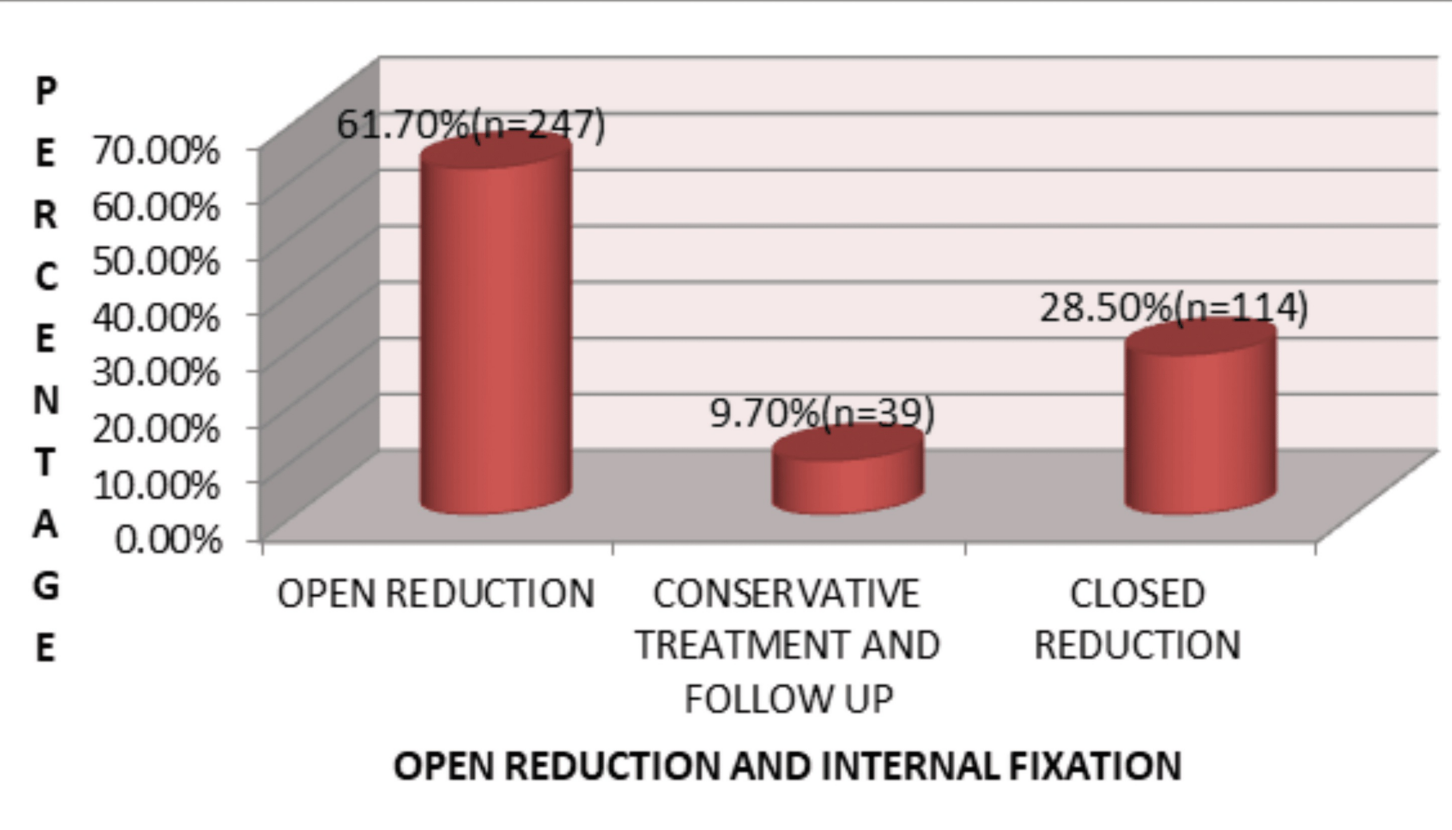
Distribution of treatment modalities for maxillofacial injuries (N = 400) Open reduction and internal fixation was performed in 61.7% (n = 247) of patients, followed by closed reduction in 28.5% (n = 114) and conservative management in 9.7% (n = 39).

Open reduction and internal fixation (ORIF) was the most commonly used treatment modality (61.7%), followed by closed reduction (28.5%) and conservative management (9.7%) (Figure 8).

## Discussion

The present study demonstrated a clear male predominance, with a male-to-female ratio of 4:1. This finding is consistent with previous reports; however, the ratio observed in the present study is comparatively lower than that reported in several other studies [5-9]. The higher incidence among males may be attributed to greater occupational and outdoor exposure, increasing susceptibility to RTAs [5,7,8]. Additionally, adolescent males are more frequently engaged in outdoor physical activities than females, further explaining the observed male predominance [6,9].

The most commonly affected age group in the present study was 21-30 years (41.5%), which is in agreement with findings reported by Kamulegeya et al. [5] and several other studies [9-12]. Individuals in this age group are typically more active socially and economically, often have limited driving experience, and are more likely to engage in risk-taking behavior, making them more vulnerable to maxillofacial injuries [10-12].

RTAs were identified as the most common etiology of maxillofacial injuries, accounting for 77.7% of cases. This observation is consistent with findings reported by Ansari [13] and several other studies [5,8,14-24]. The high prevalence of RTA-related injuries in developing countries can be attributed to inadequate enforcement of traffic regulations, poor compliance with safety measures, lack of awareness regarding protective devices, congested road conditions, overloaded vehicles, inadequate pedestrian infrastructure, and poorly maintained two-wheelers [14,15,21,22].

In contrast, falls from height were the most common cause of maxillofacial injuries among females (35%). Similar trends have been reported in previous studies, which suggest that females are less frequently involved in outdoor occupational activities and high-velocity trauma [25,26].

Two-wheelers were the most frequently involved vehicles in RTAs, corroborating findings from earlier studies [14,21,22,25]. Compared with four-wheelers, two-wheelers provide minimal protection and stability, making riders more susceptible to injury. Skidding was identified as the most common mechanism of injury, followed by rear-impact collisions [21,22].

Although protective devices such as helmets and seat belts are known to significantly reduce the incidence and severity of facial injuries, their use remains suboptimal despite mandatory legislation in India [14,21,25]. This highlights the need for improved public awareness campaigns and stricter enforcement of traffic safety regulations [13,14].

The middle third of the face was the most commonly affected region (53%), which correlates with findings reported in other studies [6,21,24]. However, several authors have reported the lower third of the face as the most frequently fractured region [5,11,13,23,27]. The mandible was the most commonly fractured bone in the present study. Despite being the strongest facial bone, its prominence, mobility, and anatomical configuration make it particularly vulnerable to traumatic forces, as reported in previous studies [5,13,14,17-19,22,27-29].

The condyle was the most frequently involved site of mandibular fracture, consistent with several studies [17,19,21,26,29]. In contrast, other investigators have reported the mandibular body [18,22,23], parasymphysis [24], or symphysis [5] as the most common fracture site. The high incidence of condylar fractures in RTA patients may be explained by inadequate protection of the chin provided by commonly used helmets, resulting in indirect transmission of impact forces to the condyle [21,25]. Angle fractures were also commonly observed, possibly due to the thinner cross-section of the bone and the presence of impacted third molars, which predispose this region to lateral impact forces [19,22].

Within the middle third of the face, the maxilla was the most commonly fractured bone, consistent with previous reports [5,8,29]. Most midface fractures occurred at the Le Fort I level, similar to findings reported by other authors [8,19]. Zygomaticomaxillary complex fractures were the most frequent midface fractures associated with RTAs, which is in agreement with earlier studies [5].

Among pediatric patients, the most affected age group was 11-20 years (8.7%), with the condyle being the most common fracture site. The condyle’s thin cortical rim and high cancellous bone content render it particularly susceptible to injury in this age group [26].

Associated injuries were frequently observed, with head injuries being the most common (63.2%), followed by lower limb injuries (12.8%). Clavicular injuries were the least common (1.7%) [25].

Management strategies varied according to fracture type, patient age, and associated injuries. Conservative management was employed for undisplaced fractures of the zygomatic arch, nasal bones, and pediatric condylar fractures. Open reduction with semi-rigid internal fixation was performed in 61.7% of cases. Treatment decisions were influenced by fracture characteristics, patient age, associated injuries, availability of treatment facilities, and socioeconomic factors [13,24,26].

These findings provide meaningful insight for improving both clinical practice and trauma care planning. The predominance of RTA-related maxillofacial injuries, especially among young male two-wheeler users, suggests that tertiary trauma centers should be consistently prepared to manage complex facial fractures that are frequently accompanied by head injuries. In such high-risk groups, prioritizing early airway stabilization, rapid diagnostic imaging, and timely surgical management can play a crucial role in improving survival and functional recovery.

From an institutional perspective, the high requirement for surgical fixation highlights the importance of maintaining adequate operative infrastructure, ensuring continuous availability of fixation systems, and supporting specialized maxillofacial trauma teams. The frequent occurrence of associated head injuries further emphasizes the need for integrated care pathways involving neurosurgery, anesthesia, and critical care services to ensure comprehensive trauma management.

In addition, these observations reinforce the value of strengthening trauma referral pathways, improving emergency response coordination, and encouraging clinician-led community education on road safety and helmet use. Implementing these measures may contribute to lowering injury severity, enhancing long-term functional and aesthetic outcomes, and promoting more efficient use of healthcare resources in tertiary trauma settings.

Limitations of the study

The findings of this investigation should be interpreted with consideration of certain constraints. As the data were collected from a single tertiary care center, the observations may not completely represent patterns seen in the general population. Inclusion of only hospital-presenting cases may have introduced sampling bias. The absence of extended follow-up restricted the evaluation of long-term functional and therapeutic outcomes. Injuries of lesser severity that did not require hospital admission might not have been captured, which could influence the overall incidence pattern. In addition, a detailed assessment of social, economic, and environmental contributors to trauma was beyond the scope of this study. Larger multicenter investigations with prospective long-term monitoring are required to obtain more comprehensive epidemiological evidence.

## Conclusions

Maxillofacial fractures predominantly affect young adult males and are most frequently caused by RTAs involving two-wheelers. The mandible, especially the condylar region, and the midfacial skeleton are the most commonly affected sites. Preventive measures, including strict traffic regulation enforcement, public education on road safety, and the use of protective headgear, are critical to reducing these injuries.

This was a single-center, hospital-based study, which may limit the generalizability of the findings. Patients with minor injuries who did not report to the tertiary care hospital were not included. Long-term functional and aesthetic outcomes could not be adequately assessed due to limited follow-up. Additionally, pre-hospital factors, detailed analysis of associated systemic injuries, and socio-behavioral factors such as alcohol use and protective device compliance were not evaluated.
